# Monogenean fauna of alien tilapias (Cichlidae) in south China

**DOI:** 10.1051/parasite/2019003

**Published:** 2019-02-04

**Authors:** Shuai Zhang, Tingting Zhi, Xiangli Xu, Yingying Zheng, Charles Félix Bilong Bilong, Antoine Pariselle, Tingbao Yang

**Affiliations:** 1 State Key Laboratory of Biocontrol, Guangdong Provincial Key Laboratory for Improved Variety Reproduction of Aquatic Economic Animals, and Research Center for Parasitic Organisms, School of Life Sciences, Sun Yat-sen University Guangzhou 510275 China; 2 Laboratory of Parasitology and Ecology, University of Yaoundé I PO Box 812 Yaoundé Cameroon; 3 ISEM, Univ Montpellier, CNRS IRD Montpellier France

**Keywords:** Tilapias, *Enterogyrus*, *Cichlidogyrus*, *Scutogyrus*, *Gyrodactylus*, China

## Abstract

Tilapias are important aquaculture fishes that have been introduced widely all over the world, often carrying their monogenean parasites with them. An extensive investigation on monogeneans of invasive tilapias was conducted in 19 natural water sources in south China between July 2015 and December 2017. We found nine known species of monogeneans, i.e., *Enterogyrus coronatus*, *E. malmbergi*, *Cichlidogyrus cirratus*, *C. halli*, *C. sclerosus*, *C. thurstonae*, *C. tilapiae*, *Scutogyrus longicornis*, *Gyrodactylus cichlidarum*, and one unknown *Gyrodactylus* species. In addition to reporting ten new hosts and four new geographical records, we observed new morphological characteristics of these species. Observation on living specimens of *Enterogyrus* spp. demonstrated that these two species have characteristic opisthaptoral retraction capacities, while the opisthaptor glands were not observed in our specimens of *E. coronatus* and *E. malmbergi*. The morphological differences of the accessory piece of the male copulatory complex between *C. cirratus* and *C. mbirizei* (character for species differentiation) could result from the observation at different perspectives, which indicates that *C. mbirizei* is likely a synonym of *C. cirratus*. A more detailed structure of the sclerotized parts of *Cichlidogyrus* spp. and *S. longicornis* were revealed by scanning electron microscopy. As was the case for the monogeneans found on alien tilapias from other geographic regions, the present study confirmed the high potential of these monogeneans to establish populations in new habitats.

## Abstract (fr)

Les tilapias sont des poissons importants pour l’aquaculture dans le monde entier, où ils ont été largement introduits, souvent accompagnés de leurs Monogènes parasites. Suite à une étude extensive menée de juillet 2015 à décembre 2017 dans le Sud de la Chine, nous avons retrouvé 9 espèces déjà connues de ces parasites, à savoir *Enterogyrus coronatus*, *E. malmbergi*, *Cichlidogyrus cirratus*, *C. halli*, *C. sclerosus*, *C. thurstonae*, *C. tilapiae*, *Scutogyrus longicornis*, *Gyrodactylus cichlidarum*, plus un *Gyrodactylus* inconnu. En plus de dix nouveaux hôtes et de quatre nouvelles localités signalés pour ces espèces, nos résultats montrent quelques différences morphologiques avec les descriptions originelles des espèces. Sur les spécimens vivants d’*Enterogyrus* l’opisthapteur montre des capacités de rétractation variables, ce qui n’a jamais été décrit chez *E. coronatus* et *E. malmbergi ;* ces hapteurs ne présentent pas de glandes. La différence principale entre *C. cirratus* et *C. mbirizei* (la forme de l’extrémité de la pièce accessoire de l’organe copulateur) pourrait n’être que le résultat de l’angle avec lequel on l’observe, ce qui pourrait indiquer une synonymie entre ces deux espèces. Des images en microscopie électronique à balayage montrent de nouveaux détails des pièces sclérifiées des espèces de *Cichlidogyrus* sp. et de *S. longicornis*. Comme cela a été le cas pour les Monogènes trouvés sur des tilapias introduits dans d’autres régions du monde, la présente étude confirme leur fort potentiel à établir des populations dans de nouveaux habitats.

## Introduction

Tilapias/Tilapia is the general name of fishes belonging to *Oreochromis* Günther, 1889, *Sarotherodon* Rüppell, 1852, *Tilapia* Smith, 1840 and *Coptodon* Gervais, 1848, all members of the Cichlidae. They are important aquaculture fishes in the world and have been introduced to at least 140 countries and have turned into worldwide invasive fishes [10]. As an important component of parasite fauna of tilapias, monogenean species reported from indigenous tilapias are very abundant, especially *Cichlidogyrus* species [26, 49]. In the course of tilapias introduction, monogenean species have unintentionally been brought to non-native countries, including the United States [40], Australia [57], Brazil [22, 56], China [28, 37, 58, 59], Colombia [25], Cuba [36], Iraq [1], Japan [33], Malaysia [29], Mexico [23, 46], Philippines [2], South Africa [26, 30], Thailand [27] and the UK [20], although there was a report of the whole gill parasite community loss [17].

As the country with the highest tilapias aquaculture production [16], China initially introduced Mozambique tilapia (*O. mossambicus* Peters, 1852) from Vietnam in 1956; other tilapias were then also introduced for culture or breed improvement [9, 60]. In the course of tilapias culture, the escapees gradually established wild populations in many natural waters of south China [21], which has become a great concern because they will not only damage the aquatic community, but also act as a refuge for aquaculture pathogens. In addition, they could acquire native parasites ([8, 23], and our unpublished data) and may spillback the parasites acquired to the endemic hosts [24]. However, extensive investigation of monogeneans of tilapias was lacking, although several sporadic reports indicated the existence of the alien gill parasites on tilapias in China [28, 37, 58, 59]. To fill this gap, an extensive investigation on the monogenean fauna of feral tilapias was carried out in south China between July 2015 and December 2017. The results presented in this paper include the monogenean fauna of tilapias and a supplementary description of new morphological features for three species.

## Material and methods

### Ethics

All the experimental handling was carried out in compliance with animal safety and ethics rule issued by the School of Life Sciences, Sun Yat-sen University.

### Host and parasite collection

**Figure 1:**
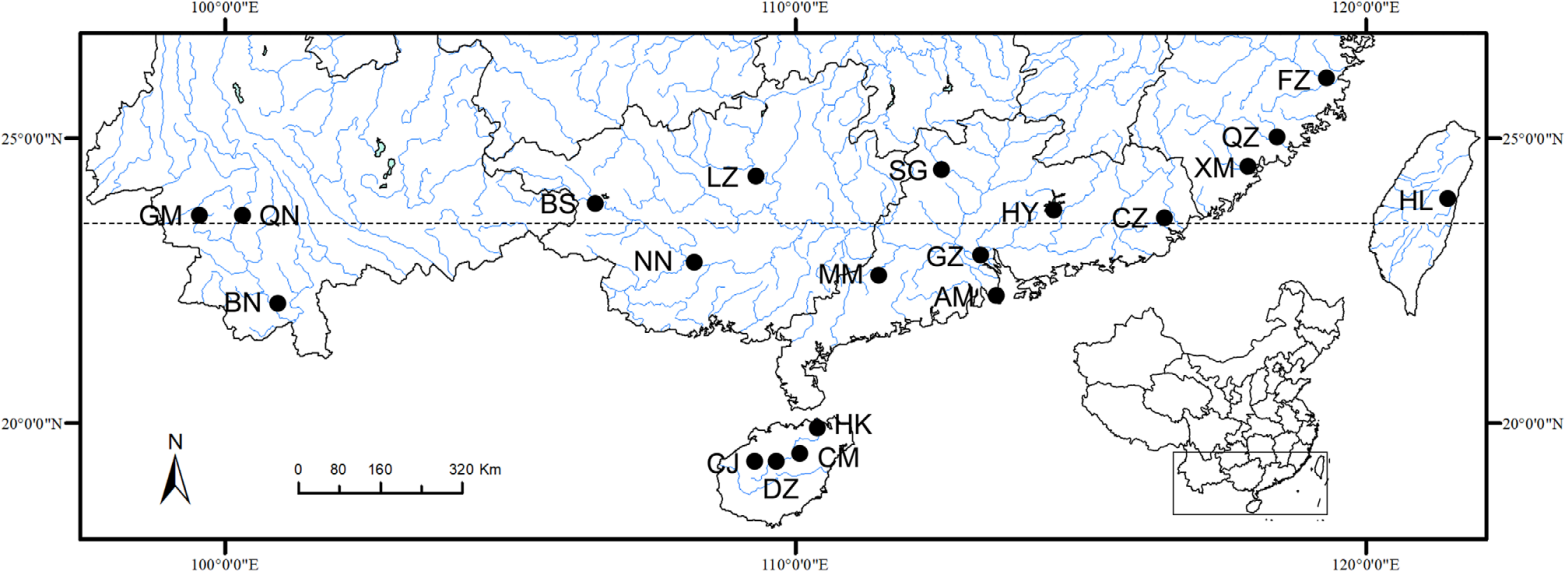
Map of investigation sites of tilapias in south China. AM natural preservation zone, Macau; BN Lancang River, Xishuangbanna, Yunnan Province; BS Boai River, Baise, Guangxi Province; CJ Shilu reservoir, Changjiang, Hainan Province; CM Jiatan reservoir, Chengmai, Hainan Province; CZ Han River, Chaozhou, Guangdong Province; DZ Songtao reservoir, Danzhou, Hainan Province; FZ Min River, Fuzhou, Fujian Province; GM Nongba reservoir, Lincang, Yunnan Province; GZ fish farm and pond in Sun Yat-sen University, Guangzhou, Guangdong Province; HK Nandu River, Haikou, Hainan Province; HL fish market, Hualien, Taiwan; HY Xinfengjiang reservoir, Heyuan, Guangdong Province; LZ Liu River, Liuzhou, Guangxi Province; MM Gaozhou reservoir, Maoming, Guangdong Province; NN Bachi River, Nanning, Guangxi Province; QN Lancang River, Lincang, Yunnan Province; QZ Jin River, Quanzhou, Fujian Province; SG Nanshui reservoir, Shaoguan, Guangdong Province; XM Xixi River, Xiamen, Fujian Province.

Investigation of wild tilapias and their monogenean fauna was conducted in 19 natural waters sources in south China. These sampling locations were selected based on field study and reports [21, 37, 59] to cover the distribution of wild tilapias in south China (Fig. 1, Table 1). Year-round investigations were implemented monthly in three reservoirs from April 2016 to August 2017: Nanshui reservoir (24°44′N, 113°10′E), Gaozhou reservoir (22°08′N, 111°05′E) and Songtao reservoir (19°24′N, 109°33′E) to cover the seasonality of infection (data about seasonality of infection unpublished). Fishes were identified by morphological features according to FishBase (www.fishbase.org). Nile tilapia *Oreochromis niloticus* and its hybrids were accepted as *O. niloticus* due to their indistinguishable morphologies. *Oreochromis niloticus* samples in Guangzhou, Guangdong Province were purchased from a local fish farm or caught from a small pond in the south campus of Sun Yat-sen University. These fishes were kept in the laboratory for observation of live parasite specimens.

**Table 1:** Monogeneans of invasive tilapias with mean abundance in south China.

Sites	Abbr.	Waters	Host species	No.	Cci	Cha	Csc	Cth	Cti	Slo	Eco	Ema
Baise	BS	River	*C. zillii*	30	0.3	0.1	×	×	0.8	×	0.3	×
			*S. galilaeus*	30	×	<0.1	0.6	×	11.4	×	×	×
			*O. niloticus*	30	2	6.6	6.1	1.6	12.9	3.4	×	0.1
Changjiang	CJ	Reservoir	*C. zillii*	20	0.9	0.1	×	×	3.4	1.3	×	×
			*O. niloticus*	22	1.5	2.3	0.1	1.4	42.7	0.9	×	×
Chengmai	CM	Reservoir	*C. zillii*	128	0.9	0.2	×	<0.1	5.1	<0.1	×	×
			*O. niloticus*	39	0.1	3.5	2.5	3	9.8	6.1	×	<0.1
Chaozhou	CZ	River	*C. zillii*	108	0.1	×	<0.1	×	0.4	<0.1	×	×
			*S. galilaeus*	13	×	×	0.4	×	6.5	×	×	0.2
			*O. niloticus*	26	0.3	×	1.2	×	7.1	1.3	×	0.1
Danzhou	DZ	Reservoir	*C. zillii*	23	×	×	×	×	0.4	×	×	×
			*O. mossambicus*	15	0.3	0.1	8.9	0.4	5.5	1.3	×	1.6
			*O. niloticus*	492	5.2	7.7	3.7	7.5	11.9	6.9	×	1.6
Fuzhou	FZ	River	*C. zillii*	25	×	×	×	×	1	×	–	–
			*S. galilaeus*	13	×	×	0.2	×	1.5	×	–	–
			*O. niloticus*	17	×	×	0.1	×	0.6	×	–	–
Gengma	GM	Reservoir	*O. niloticus*	31	0.6	×	1.3	×	5.9	2.9	×	×
Guangzhou	GZ	Captivity	*O. niloticus*	>50	×	×	√	√	√	√	×	√
Haikou	HK	Estuary	*C. zillii*	10	×	×	×	×	4.8	×	×	×
			*O. mossambicus*	32	×	×	×	×	×	×	×	×
			*O. niloticus*	32	×	×	×	×	1	×	×	×
Heyuan	HY	Reservoir	*C. zillii*	29	<0.1	×	×	×	3.7	×	0.2	×
			*S. galilaeus*	6	×	×	×	×	6.3	×	×	×
			*O. niloticus*	7	2	3.4	0.7	0.3	26.9	2.1	×	×
Hualien	HL	Captivity	*O. niloticus*	1	1	×	×	1	1	1	–	–
Liuzhou	LZ	River	*C. zillii*	40	×	×	×	×	0.3	×	1.8	×
			*O. niloticus*	13	×	0.3	1.4	×	4.1	1.2	0.4	0.5
Macau	AM	Estuary	*C. zillii*	6	×	×	×	×	×	×	×	×
			*O. niloticus*	6	×	×	×	×	×	×	×	×
Maoming	MM	Reservoir	*C. zillii*	553	0.6	0.3	<0.1	0.1	4	0.1	0.8	<0.1
			*O. niloticus*	599	2.5	5	1	4.6	15.6	5	×	0.3
Nanning	NN	River	*C. zillii*	30	<0.1	0.1	×	×	5.1	×	0.1	×
			*S. galilaeus*	6	×	×	0.3	×	14.2	×	×	×
			*O. niloticus*	32	0.4	6	4.4	4.1	19.6	10.4	×	0.1
Quannei	QN	River	*C. zillii*	34	0.7	×	×	×	3.8	0.1	3	×
			*O. niloticus*	30	2.2	×	1.2	0.8	9.5	3.8	×	1
Quanzhou	QZ	River	*C. zillii*	42	×	×	<0.1	×	0.3	×	<0.1	×
			*S. galilaeus*	26	×	×	0.7	×	7.1	×	×	×
			*O. niloticus*	31	0.1	×	2.7	0.3	4.5	1.1	×	0.2
Shaoguan	SG	Reservoir	*C. zillii*	683	<0.1	×	×	<0.1	1	×	1.8	×
Xiamen	XM	River	*C. zillii*	5	×	×	×	×	2	×	–	–
			*S. galilaeus*	19	×	×	0.1	×	0.6	×	–	–
			*O. niloticus*	28	×	×	1.5	0.6	5	1.2	–	–
Xishuangbanna	BN	River	*C. zillii*	23	×	×	×	×	0.2	<0.1	1	×
			*O. niloticus*	42	0.1	5.6	3.5	1.7	2	1.5	×	0.1

Abbr., abbreviation of sites; No., sampling number; Cti, *Cichlidogyrus tilapiae*; Cci, *C. cirratus*; Cth, *C. thurstonae*; Csc, *C. sclerosus*; Cha, *C. halli*; Slo, *Scutogyrus longicornis*; Eco, *E. coronatus*; Ema, *E. malmbergi*. √, sampled, but data not recorded; ×, not sampled; –, not examined. There were twelve *G. cichlidarum* sampled, including two *G. cichlidarum* collected from Gaozhou reservoir, Maoming (MM) and Songtao reservoir, Danzhou (DZ), respectively, and eight collected from laboratory reared *O. niloticus* in Guangzhou (GZ). Only one *Gyrodactylus* sp1. collected from Songtao reservoir, Danzhou.

Fish samples caught in the wild were individually killed and examined for parasites in the gills, stomach and urinary bladder. The parasite examination for the fish samples kept in the laboratory included the body surface. For identification, live monogeneans were detached with a dissecting needle, pipetted out, and mounted in a drop of ammonium picrate glycerin (GAP) on a slide under a coverslip, which was sealed using nail polish. After identification, target slides were rinsed in distilled water for 12–24 h until the nail polish could be easily removed, and the detached specimens were stored in vessels for further studies.

### Light microscopy and scanning electron microscopy

For SEM studies, worms were processed for scanning electron microscopy (SEM) according to Mo and Appleby [38] or Fannes et al. [15], sputter coated with gold and finally examined under Quanta 400 (FEI, Netherlands) in the Instrumental Analysis & Research Center, Sun Yat-sen University. For light microscopy studies, identified worms were digested following the protocol depicted by Fannes et al. [15], and later remounted in GAP on slides. Or alternatively, specimens were rinsed several times with water before being stained in Modified Gomori’s Trichrome, dehydrated in an ethanol gradient, cleared in clove oil, and finally mounted in neutral Canada balsam. For *Gyrodactylus* species, GAP preserved specimens were digested *in situ*: proteinase K solution was dripped on one side of the coverslip, while a piece of filter paper was placed on the opposite side until the GAP solution was entirely replaced by proteinase K solution; later the same method was used to replace the digestive fluid by GAP solution.

### Morphological analyses

**Figure 2:**
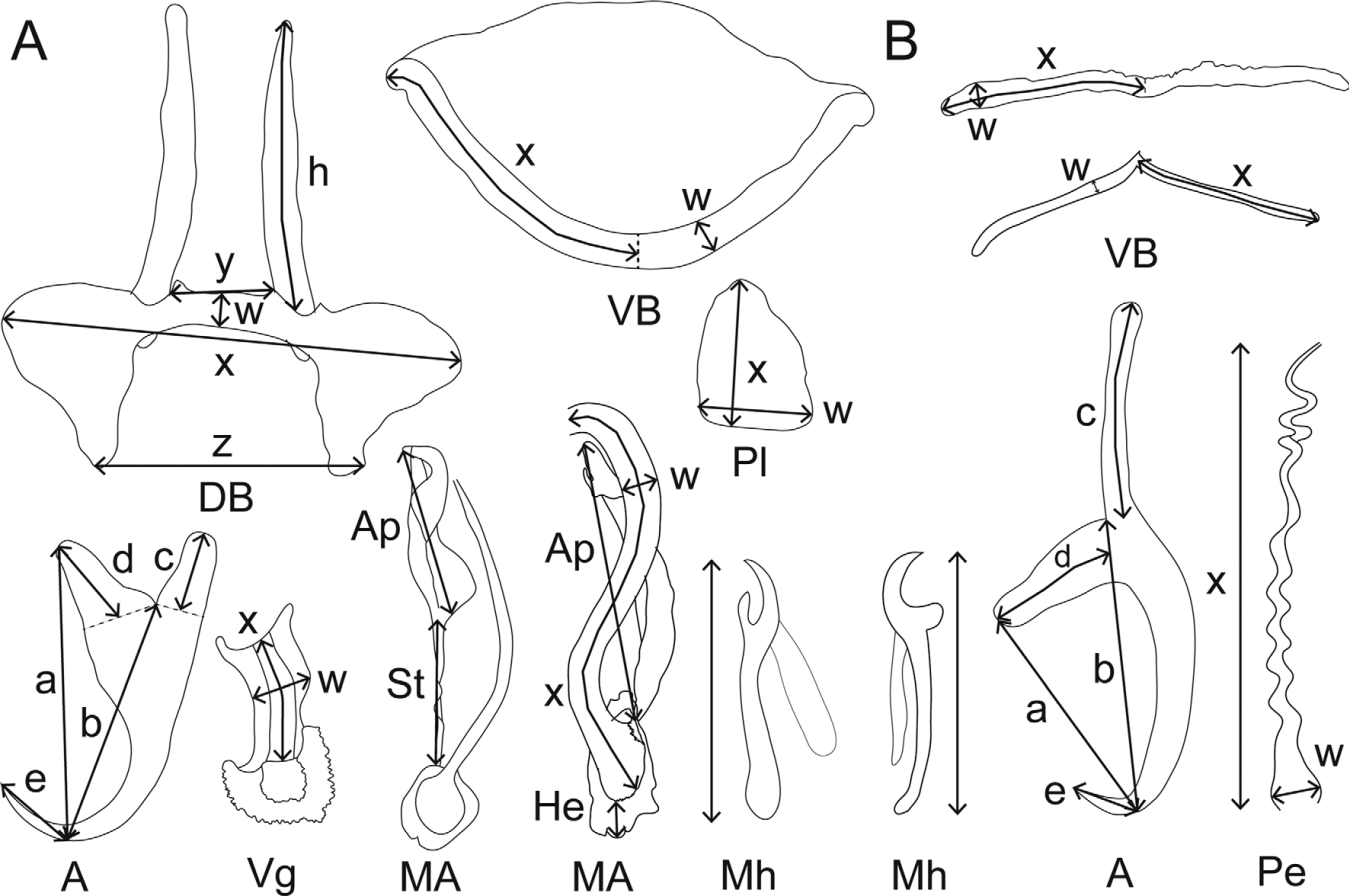
(A–B) Measurements studied. (A) Measurements used to study *Cichlidogyrus* spp. and *Scutogyrus* spp. DB dorsal bar: *h*, length of dorsal bar auricle; *w*, dorsal bar maximum width; *x*, dorsal bar total length; *y*, distance between auricles; *z*, dorsal bar base end length. A anchor: *a*, anchor total length; *b*, anchor blade length; *c*, anchor shaft length; *d*, anchor guard length; *e*, anchor point length. Vg vagina: *x*, vagina total length, *w*, vagina maximum width. MCC male copulatory complex: Ap, accessory piece straight length; St, stalk length; He, heel straight length; Pe, penis: *x*, penis total curved length; *w*, penis maximum width. Pl auxiliary plate: *x*, auxiliary plate total length; *w*, auxiliary plate maximum width. H hook straight length. VB ventral bar: *w*, ventral bar maximum width; *x*, length of one ventral bar branch. (B) Measurements used to study *Enterogyrus* spp. VB ventral bar: *x*, length of one ventral bar branch; *w*, ventral bar maximum width. A anchor: a, anchor total length; *b*, anchor blade length; *c*, anchor shaft length; *d*, anchor guard length; *e*, anchor point length. Pe penis: *x*, penis total curved length; *w*, penis base maximum width.

The numbering of the sclerotized parts of the species in genera *Scutogyrus*, *Cichlidogyrus* and *Enterogyrus* was adopted from ICOPA IV [14] and the terminology followed Pariselle and Euzet [49]. For *Gyrodactylus* species, the measurements of sclerotized parts and terminology followed Shinn et al. [54]. The terminology was employed as follows: anchor instead of gripus or hamulus; hooks rather than marginal hook, uncinulus or hooklet, and ventral bar instead of ventral transverse bar. Additionally, the ventral bar length represents the length of one branch rather than the whole length (with that of *Gyrodactylus* as an exception). The metrics are shown in Figure 2.

Photographs and measurements of the sclerotized parts were taken under an Olympus DX41 microscope equipped a DP73 Olympus camera (Olympus, Japan), and processed by the software cellSens Standard 1.7.1. Illustrations were drawn freehand with the aid of an Olympus U-DA drawing attachment and then digitized and processed using Adobe Illustrator CS6 (Adobe, USA). All measurements were taken in micrometers and presented in the following order: mean ± standard deviation (minimum – maximum, number of measurements). Voucher specimens were stored in the Research Center for Parasitic Organisms, School of Life Sciences, Sun Yat-sen University (SYSU) and a set of whole-mount specimens was also deposited in the Muséum National d’Histoire Naturelle, France (MNHN).

## Results

A total of 3,426 feral tilapias (including 1,789 *Coptodon zillii*, 113 *Sarotherodon galilaeus*, 1,477 *O. niloticus* and 47 *O. mossambicus*) and more than 50 cultured *O. niloticus* from south China were examined for monogenean parasites (see Table 1). The ten species of monogeneans collected belong to two families:

- Ancyrocephalidae Bychowsky & Nagibina, 1968 with three genera *Enterogyrus* Paperna, 1963, *Cichlidogyrus* Paperna, 1960 and *Scutogyrus* Pariselle & Euzet, 1995.
- From the stomach of the fish:

*Enterogyrus coronatus* Pariselle, Lambert & Euzet, 1991 and *E. malmbergi* Bilong Bilong, 1988.
From gills of the fish:
*Cichlidogyrus cirratus* Paperna, 1964; *C. halli* Price & Kirk, 1967; *C. sclerosus* Paperna & Thurston, 1969; *C. thurstonae* Ergens, 1981 and *C. tilapiae* Paperna, 1960.
*Scutogyrus longicornis* Paperna & Thurston, 1969.

- Gyrodactylidae Cobbold, 1864 with the genus *Gyrodactylus* von Nordmann, 1832 and the species *G. cichlidarum* Paperna, 1968 and *Gyrodactylus* sp1.

The urinary bladders did not host monogenean species.

### Family Ancyrocephalidae Bychowsky & Nagibina, 1968

#### Genus *Enterogyrus* Paperna, 1963

##### *Enterogyrus coronatus* Pariselle, Lambert & Euzet, 1991

Type host: *Coptodon guineensis* (Perciformes: Cichlidae).

Hosts: *Oreochromis niloticus* and *Coptodon zillii*.

Site of infection: Stomach.

Type locality: Ebrié lagoon, Cote d’Ivoire.

Localities: Nanshui reservoir, Shaoguan, Guangdong province; Gaozhou reservoir, Maoming, Guangdong province; Xinfengjiang reservoir, Heyuan, Guangdong Province; River Liu, Liuzhou, Guangxi province; Boai River, Baise, Guangxi Province; Bachi River, Nanning, Guangxi Province; Jin River, Quanzhou, Fujian Province; Lancang River, Lincang, Yunnan Province; Lancang River, Xishuangbanna, Yunnan Province.

Number of voucher specimens observed: 31 (SYSUECO1–30; MNHN HEL906).

**Figure 3:**
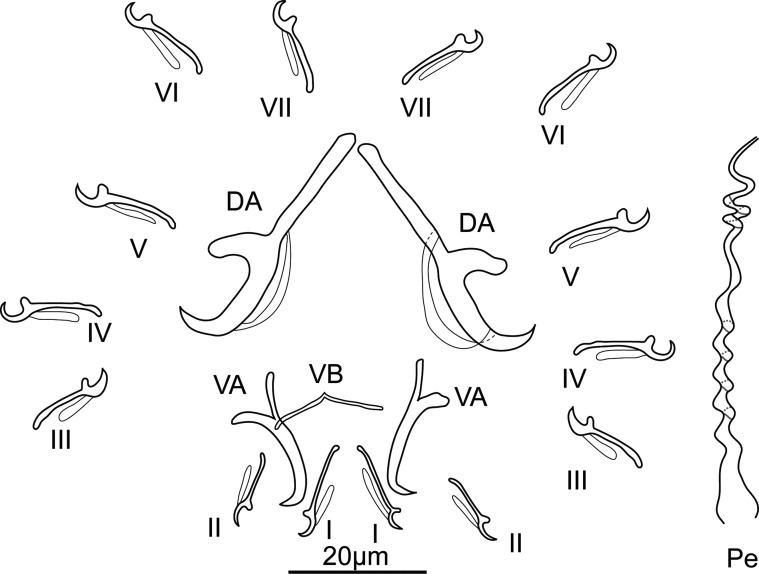
Drawings of sclerotized parts of *E. coronatus* Pariselle, Lambert & Euzet, 1991. DA, dorsal anchor; VA, ventral anchor; VB, ventral bar; Pe, penis; I–*VII*, hooks.

**Figure 4:**
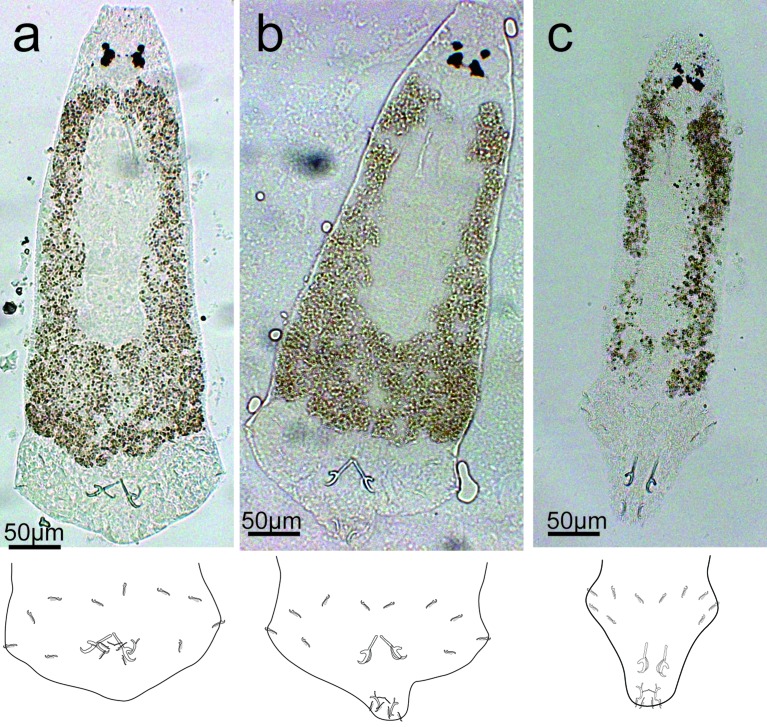
Different body shape of *E. coronatus* Pariselle, Lambert & Euzet, 1991 under coverslip and the motion of opisthaptoral sclerotized parts depicted in drawings. (a) Cup-shaped body (retracted); (b) interim body shape; (c) tongue-shaped body (relaxed).

Description (Figs. 3, 4a–c, 10j): Four eyespots well developed without lenses. Tegument thick and transversally striated. Opisthaptor shape variable (tongue- or cup-shaped). Adults 421 ± 76.2 (292–574, 30) long and 149 ± 26.7 (101–205, 30) wide. Pharynx globular 32 ± 6.9 (22–56, 30) in diameter. Dorsal anchor with shaft longer than blade: *a* = 10 ± 2 (8–20, 29), *b* = 14 ± 2.6 (10–27, 29), *c* = 18 ± 3 (13–30, 29), *d* = 7 ± 1.2 (5–13, 29), *e* = 5 ± 1.3 (3–9, 29). Ventral anchor smaller than dorsal one: *a* = 14 ± 2 (9–23, 29), *b* = 12 ± 1.6 (9–20, 29), *c* = 5 ± 1 (4–10, 29), *d* = 5 ± 1.1 (3–9, 29), *e* = 4 ± 0.9 (2–7, 28). Thin and weak V-shaped ventral bar: *x* = 9 ± 2.3 (7–16, 23), *w* = 1 ± 0.2 (0–1, 23). Hooks (marginal hooks) robust except first and second pairs (thinner): I = 12 ± 0.9 (11–13.7, 25), *II* = 12 ± 0.8 (10–13.1, 25), *III* = 13 ± 0.9 (10–13.8, 23), *IV* = 13 ± 0.9 (10–14.3, 27), *V* = 14 ± 0.9 (11–15.7, 28), VI = 14 ± 0.7 (12–14.7, 27), VII = 13 ± 0.7 (12–14.8, 27). Penis spiral pattern: 4–2–3, 52 ± 13.5 (46–123, 30) long and 6 ± 1.7 (3–13, 30) wide at the base. Eggs oval, length 72 (*n* = 1) and width 66 (*n* = 1).

Remarks: According to the measurements and descriptions of the sclerotized parts, the present specimens resemble *E. coronatus* Pariselle et al. [50]. The hooks were almost identical in length but pairs I and II are discernibly slenderer than others [30]. The parasite can stretch out its retractable opisthaptor to anchor the stomach wall and withdraw it to relocate to a new site during movement (based on the observations *in situ* and GAP preserved specimens). As a result, *E. coronatus* can present two body shapes depending on the different status of opisthaptor: cup- or tongue-shaped (Fig. 4). No opisthaptor glands were observed in the stained specimens, which was different from the description of *E. cichlidarum* by Paperna [42].

In the eight locations (Baise, Heyuan, Liuzhou, Maoming, Nanning, Quannei, Quanzhou and Xishuangbanna) where *E. coronatus* coexisted with the hosts *O. niloticus* and *C. zillii*, *C. zillii* was always found with *E. coronatus* infection, while *O. niloticus* was only found to be infected in Liuzhou (see Table 1). Especially in Maoming, where both *O. niloticus* and *C. zillii* were monthly sampled for year-round, *E. coronatus* was exclusively collected from *C. zillii* (prevalence: 32.2%; mean intensity: 2.1). In the whole investigation, *E. coronatus* was not collected from *S. galilaeus* and *O. mossambicus*. This species had previously been reported from *Tilapia guineensis* (*Coptodon guineensis*) [50], *Tilapia dageti* (*Coptodon dageti*) [34], and *Pseudocrenilabrus philander philander* [30]. In a word, *E. coronatus* shows host preference to *C. zillii* in China and it possesses the potential to infect other cichlids. The occurrence of this species in the stomach of *O. niloticus* and *C. zillii* from China provides new localities and new host records.

##### *Enterogyrus malmbergi* Bilong Bilong, 1988

Type host: *Oreochromis niloticus*.

Hosts: *Sarotherodon galilaeus*, *Oreochromis mossambicus*, *Oreochromis niloticus* and *Coptodon zillii*.

Site of infection: Stomach.

Type locality: Sanaga River, Cameroon.

Localities: A pond in Sun Yat-sen University and a fish farm in Guangzhou, Guangdong province; Gaozhou reservoir, Maoming, Guangdong province; Han River, Chaozhou, Guangdong Province; Boai River, Baise, Guangxi Province; Bachi River, Nanning, Guangxi Province; Liu River, Liuzhou, Guangxi Province; Songtao reservoir, Danzhou, Hainan province; Jiatan reservoir, Chengmai, Hainan Province; Jin River, Quanzhou, Fujian Province; Lancang River, Xishuangbanna, Yunnan Province; Lancang River, Lincang, Yunnan Province.

Voucher specimens observed and deposited: 32 (SYSUEMA1–31; MNHN HEL907).

**Figure 5:**
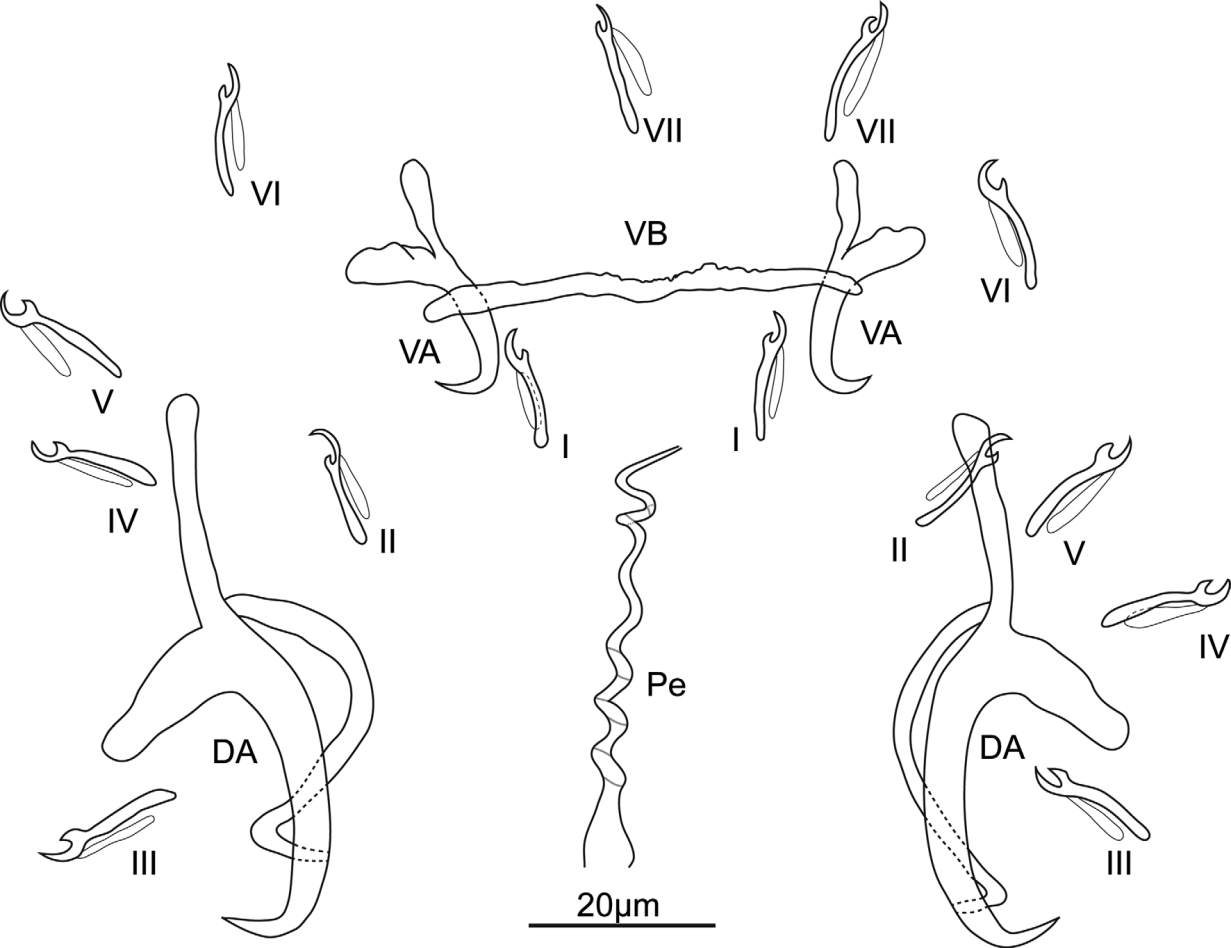
Drawings of sclerotized parts of *E. malmbergi* Bilong Bilong, 1988. DA, dorsal anchor; VA, ventral anchor; VB, ventral bar; Pe, penis; I–VII, hooks.

**Figure 6:**
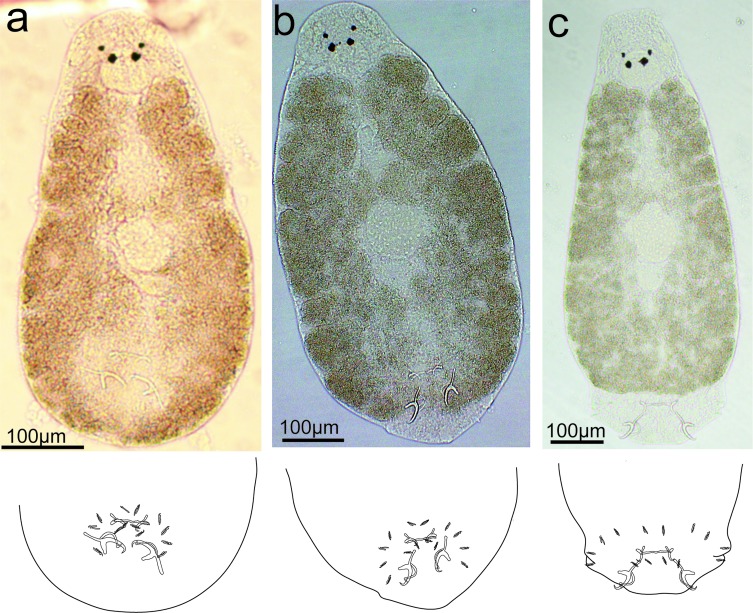
Different body shape of *E. malmbergi* Bilong Bilong, 1988 under coverslip and the motion of opisthaptoral sclerotized parts depicted in drawings. (a) Cup-shaped body (retracted); (b) interim body shape; (c) cup-shaped body (relaxed).

Description (Figs. 5, 6a–c, 10i): Four eyespots well developed without lenses. Tegument thick and striated transversally. Opisthaptor slightly retractable, body cup-shaped. Adults 721 ± 94.2 (481–854, 31) long and 284 ± 43.3 (218–361, 31) wide. Pharynx globular 56 ± 10.3 (38–77, 31) in diameter. Dorsal anchor with shaft shorter than blade: *a* = 26 ± 1.3 (23–29, 31), *b* = 36 ± 1.1 (33–38, 31), *c* = 26 ± 1.4 (22–29, 31), *d* = 16 ± 1.1 (13–18, 31), *e* = 7 ± 0.7 (5–8, 31). Ventral anchor smaller than dorsal one: *a* = 20 ± 1 (18–22, 31), *b* = 15 ± 0.6 (14–16, 31), *c* = 10 ± 0.9 (7–12, 31), *d* = 10 ± 0.8 (7–11, 31), *e* = 5 ± 0.7 (4–7, 31). Straight-shaped robust ventral bar: *x* = 25 ± 1.5 (21–28, 31), *w* = 3 ± 0.4 (2–4, 31). Hooks short, robust and basically identical: I = 15 ± 1.1 (13–18, 29), II = 14 ± 1 (12–17, 29), *III* = 14 ± 0.7 (13–15, 29), IV = 15 ± 1.2 (12–17, 29), *V* = 15 ± 1 (13–17, 29), VI = 15 ± 1.1 (12–17, 29), VII = 15 ± 0.8 (14–17, 30). Penis spiral pattern: 3–2–1/3–1–2, 49 ± 3.9 (43–58, 31) long and 6 ± 0.8 (5–8, 31) wide at the base. Eggs oval, length 91 ± 2.8 (86–93, 5) and width 77 ± 2.7 (72–79, 5).

Remarks: The morphologies and measurements of the sclerotized parts of our specimens basically agree with the previous description of *E. malmbergi*, but are slightly larger [7], probably influenced by environmental conditions [11]. The *in situ* observation of *E. malmbergi* found that the wound in the stomach of hosts was larger than those caused by *E. coronatus* [31] and the persistence of infection will enlarge the wound. No opisthaptor glands were observed in this species, which was consistent with descriptions of other *Enterogyrus* species [3, 5–7, 30, 50] except *E. cichlidarum* which was ever described with opisthaptor glands [42]. In addition, *E. malmbergi* has a much less retractable opisthaptor (only cup-shaped) than *E. coronatus* (Figs. 4, 6).

In the ten sampling sites (Baise, Chengmai, Chaozhou, Danzhou, Liuzhou, Maoming, Nanning, Quannei, Quanzhou and Xishuangbanna), where *E. malmbergi* coexisted with the hosts *O. niloticus* and *C. zillii*, this parasite was always collected from *O. niloticus*, but not from *C. zillii* except in Maoming where both *O. niloticus* and *C. zillii* were found to be infected (see Table 1). Even in Maoming, year-round investigations revealed that *E. malmbergi* had much higher infection levels in *O. niloticus* (prevalence: 16.4%; mean intensity: 1.4) than in *C. zillii* (prevalence: 1.4%; mean intensity: 1.1). In addition, *E. malmbergi* was also sampled from *S. galilaeus* and *O. mossambicus* in the present study, and had previously been reported from *Cichlasoma callolepis* [23]. These results indicate that *E. malmbergi* presents host preference to *O. niloticus* in China, but has the potential to infect other cichlids. It is the first record of *E. malmbergi* in China, and with *C. zillii*, *S. galilaeus* and *O. mossambicus* as new host records.

#### Genus *Cichlidogyrus* Paperna, 1960

##### *Cichlidogyrus cirratus* Paperna, 1964

Type host: *Sarotherodon galilaeus*.

Hosts: *Oreochromis mossambicus*, *Oreochromis niloticus* and *Coptodon zillii*.

Site of infection: Gills.

Type locality: Tiberias Lake, Israel.

Localities: Nanshui reservoir, Shaoguan, Guangdong province; Gaozhou reservoir, Maoming, Guangdong province; Xinfengjiang reservoir, Heyuan, Guangdong Province; Han River, Chaozhou, Guangdong Province; Boai River, Baise, Guangxi Province; Bachi River, Nanning, Guangxi Province; Songtao reservoir, Danzhou, Hainan province; Shilu reservoir, Changjiang, Hainan Province; Jiatan reservoir, Chengmai, Hainan Province; Jin River, Quanzhou, Fujian Province; Lancang River, Xishuangbanna, Yunnan Province; Nongba reservoir, Lincang, Yunnan Province; Lancang River, Lincang, Yunnan Province.

Voucher specimens observed and deposited: 31 (SYSUCCI1–30; MNHN HEL902).

**Figure 7:**
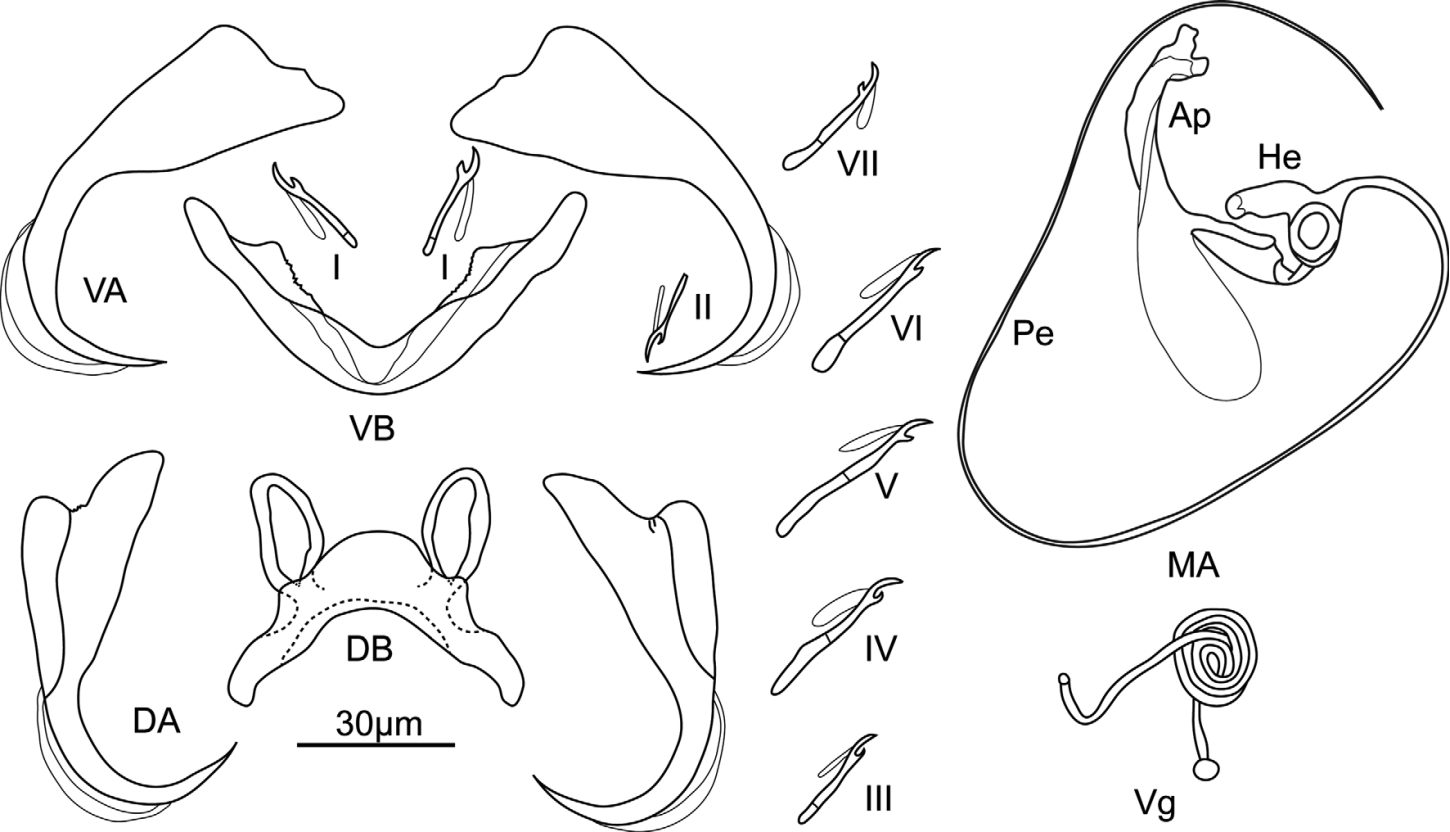
Opisthaptoral and genital sclerotized parts of *Cichlidogyrus cirratus* Paperna, 1964. Ap, accessory piece; DB, dorsal bar; DA, dorsal anchor; He, heel; Pe, penis; VA, ventral anchor; VB, ventral bar; Vg, vagina; I–VII, hooks.

**Figure 8:**
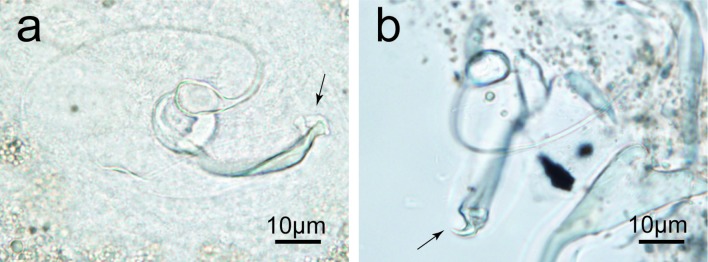
Morphology of the male copulatory complex of the same specimen of *Cichlidogyrus cirratus* observed in different angles. (a) undigested *C. cirratus*, (b) digested *C. cirratus*.

Description (Figs. 7, 8a, b, 10a, b): Adults 809 ± 199 (362–1088, 30) long and 129 ± 21.2 (89–180, 30) wide at level of ovary. Pharynx globular 31 ± 5 (21–39, 27) in diameter. Dorsal anchor with short shaft and regularly curved blade: *a* = 46 ± 1.9 (42–50, 30), *b* = 39 ± 1.4 (36–41, 30), *c* = 3 ± 1.2 (1–6, 30), *d* = 12 ± 2.4 (7–17, 30), *e* = 14 ± 1.4 (11–17, 30). Arched dorsal bar: *h* = 16 ± 1.3 (14–19, 29), *w* = 9 ± 1.4 (5–13, 30), *x* = 41 ± 2.4 (36–45, 30), *y* = 14 ± 1.5 (11–18, 28). Ventral anchor with undeveloped shaft: *a* = 49 ± 2.1 (44–52, 30), *b* = 45 ± 2.3 (40–48, 30), *c* = 1 ± 0.9 (0–3, 30), *d* = 9 ± 1.5 (6–12, 30), *e* = 17 ± 0.8 (15–18, 30). V-shaped ventral bar: *x* = 37 ± 2 (32–41, 30), *w* = 6 ± 0.6 (5–7, 30). Hooks short: I = 15 ± 0.6 (14–17, 29), II = 13 ± 0.9 (11–15, 24), *III* = 16 ± 1.2 (14–20, 26), IV = 22 ± 1.3 (19–24, 29), *V* = 24 ± 1.3 (21–27, 28), VI = 22 ± 1.8 (15–23, 28), VII = 18 ± 1 (15–20, 27). Very long and thin coiled penis, starting in a bulb with marked heel. Accessory piece, connected with the penis bulb by a rod, coated by a large, oval and thin membrane extended to the bifurcate ends: Pe = 210 ± 20.9 (165–240, 26), He = 12 ± 3.4 (8–25, 30), Ap = 41 ± 3.3 (35–49, 30). Penis end double sharped, not blunt. Very long and thin spirally coiled vagina, no valuable length could be taken.

Remarks: *Cichlidogyrus cirratus* was first described by Paperna [43] from the gills of *Tilapia galilaea* (*Sarotherodon galilaeus*) in Lake Tiberias, Israel, and redescribed by Ergens [13] from the gills of *Tilapia nilotica* (*Oreochromis niloticus*) in River Nile, Egypt. In the present study, *C. cirratus* was collected from *O. mossambicus* (new host), *O. niloticus* and *Coptodon zillii*, but was not found in its type host (*S. galilaeus*).

The long winding penis and the short first pair of hooks differentiates *C. cirratus* from all other congeneric species from cichlid hosts, except *C. mbirizei* Muterezi Bukinga et al., 2012 [39, 49]. The measurements of *C. cirratus* were consistent with those of *C. mbirizei*, and the latter was distinguished from *C. cirratus* in the original description by the shape of the accessory piece of the male copulatory complex (*C. mbirizei* without long expansion at mid-length and with two ends of rounded outgrowth *versus C. cirratus* with long expansion and hooked ends) and the vagina (double pitch in *C. mbirizei versus* sinuous in *C. cirratus*). However, the thin, long and transparent expansion in the middle of the accessory piece of *C. cirratus* was variable due to the different perspectives (Fig. 8). Moreover, two types of accessory piece extremity (hooked *versus* rounded outgrowth) could transform in digested specimens, when the accessory piece turns over (Fig. 8). These morphological features suggest that *C. mbirizei* and *C. cirratus* are likely synonymous. Pending genetic study, these two species are kept valid in the present study.

*Cichlidogyrus cirratus* (or *C. mbirizei*) was also recorded from non-native tilapias in Malaysia and Thailand [27, 29]. In addition, *Cichlidogyrus* sp. (named as *C. bananensis* by Xiao [59]) found in Lancang River in China was likely a misidentified *C. cirratus* as it shows close morphological and morphometric similarities to the latter, which was also collected from the same locality in the present study.

##### *Cichlidogyrus halli* Price & Kirk, 1967

Type host: *Oreochromis shiranus*.

Hosts: *Sarotherodon galilaeus*, *Oreochromis mossambicus*, *Oreochromis niloticus* and *Coptodon zillii*.

Site of infection: Gills.

Type locality: Upper Shire River, Malawi.

Localities: Gaozhou reservoir, Maoming, Guangdong province; Xinfengjiang reservoir, Heyuan, Guangdong Province; Boai River, Baise, Guangxi Province; Bachi River, Nanning, Guangxi Province; Liu River, Liuzhou, Guangxi Province; Songtao reservoir, Danzhou, Hainan province; Jiatan reservoir, Chengmai, Hainan Province; Shilu reservoir, Changjiang, Hainan Province; Lancang River, Xishuangbanna, Yunnan Province; Hualien, Taiwan.

Voucher specimens observed and deposited: 31 (SYSUCHA1–30; MNHN HEL903).

Remarks: The morphologies and measurements of the voucher specimens in the present study agree with the previous ones from *C. halli* [12]. This species was also described from non-native tilapias in Brazil [22], South Africa [32], Thailand [27], Malaysia [29], Japan [33] and China [37, 59]. It is the first record of *C. halli* in *Coptodon zillii* and *O. mossambicus*.

##### *Cichlidogyrus sclerosus* Paperna & Thurston, 1969

Type host: *Oreochromis mossambicus*.

Hosts: *Sarotherodon galilaeus*, *Oreochromis mossambicus*, *Oreochromis niloticus* and *Coptodon zillii*.

Site of infection: Gills.

Type locality: Kajansi, Uganda.

Localities: A pond in Sun Yat-sen University and a fish farm in Guangzhou, Guangdong province; Gaozhou reservoir, Maoming, Guangdong province; Xinfengjiang reservoir, Heyuan, Guangdong Province; Han River, Chaozhou, Guangdong Province; Boai River, Baise, Guangxi Province; Bachi River, Nanning, Guangxi Province; Liu River, Liuzhou, Guangxi Province; Songtao reservoir, Danzhou, Hainan province; Shilu reservoir, Changjiang, Hainan Province; Jiatan reservoir, Chengmai, Hainan Province; Xixi River, Xiamen, Fujian Province; Jin River, Quanzhou, Fujian Province; Min River, Fuzhou, Fujian Province; Lancang River, Xishuangbanna, Yunnan Province; Nongba reservoir, Lincang, Yunnan Province; Lancang River, Lincang, Yunnan Province.

Voucher specimens observed and deposited: 31 (SYSUCSC1-30; MNHN HELxxxx).

Remarks: The morphologies and measurements of specimens in the present study agree with the previous ones of *C. sclerosus* [12, 45]. This species has been reported from non-native tilapias in Iraq [1], Mexico [23, 46], Colombia [25], Thailand [27], Malaysia [29], South Africa [32], Brazil [22], Japan [33] and China [28, 37, 58, 59]. *Sarotherodon galilaeus* represents a new host record of this parasite.

##### *Cichlidogyrus thurstonae* Ergens, 1981

Type host: *Oreochromis niloticus*.

Hosts: *Sarotherodon galilaeus*, *Oreochromis mossambicus*, *Oreochromis niloticus* and *Coptodon zillii*.

Site of infection: Gills.

Type locality: Nile River, Egypt.

Localities: A pond in Sun Yat-sen University and a fish farm in Guangzhou, Guangdong province; Nanshui reservoir, Shaoguan, Guangdong province; Gaozhou reservoir, Maoming, Guangdong province; Xinfengjiang reservoir, Heyuan, Guangdong Province; Boai River, Baise, Guangxi Province; Bachi River, Nanning, Guangxi Province; Songtao reservoir, Danzhou, Hainan province; Shilu reservoir, Changjiang, Hainan Province; Jiatan reservoir, Chengmai, Hainan Province; Xixi River, Xiamen, Fujian Province; Jin River, Quanzhou, Fujian Province; Lancang River, Xishuangbanna, Yunnan Province; Lancang River, Lincang, Yunnan Province.

Voucher specimens observed and deposited: 32 (SYSUCTH1–31; MNHN HEL904).

Remarks: The morphologies and measurements agree with the previous ones of *C. thurstonae* [13, 47]. This species has been reported from non-native tilapias in Thailand [27], Malaysia [29], Brazil [22] and China [28, 37]. This is the first record of *C. thurstonae* from *Coptodon zillii*.

##### *Cichlidogyrus tilapiae* Paperna, 1960

Type host: *Sarotherodon galilaeus*.

Hosts: *Sarotherodon galilaeus*, *Oreochromis mossambicus*, *Oreochromis niloticus* and *Coptodon zillii*.

Site of infection: Gills.

Type locality: Jordan and coastal system, Israel.

Localities: A pond in Sun Yat-sen University and a fish farm in Guangzhou, Guangdong Province; Xinfengjiang reservoir, Heyuan, Guangdong Province; Han River, Chaozhou, Guangdong Province; Gaozhou reservoir, Maoming, Guangdong Province; Nanshui reservoir, Shaoguan, Guangdong Province; Boai River, Baise, Guangxi Province; Liu River, Liuzhou, Guangxi Province; Bachi River, Nanning, Guangxi Province; Shilu reservoir, Changjiang, Hainan Province; Jiatan reservoir, Chengmai, Hainan Province; Songtao reservoir, Danzhou, Hainan Province; Nandu River, Haikou, Hainan Province; Min River, Fuzhou, Fujian Province; Jin River, Quanzhou, Fujian Province; Xixi River, Xiamen, Fujian Province; Lancang River, Xishuangbanna, Yunnan Province; Nongba reservoir, Lincang, Yunnan Province; Lancang River, Lincang, Yunnan Province; Hualien, Taiwan.

Voucher specimens observed and deposited: 33 (SYSUCTI1–32; MNHN HEL905).

Remarks: The morphologies and measurements of the present specimens agree with the previous ones of *C. tilapiae* [12, 13, 41], which has been reported from non-native tilapias in Iraq [1], Mexico [23], Colombia [25], Thailand [27], Malaysia [29], Japan [33], South Africa [32], Australia [57] and Brazil [22, 56]. *Cichlidogyrus haplochromii* Paperna & Thurston, 1969 found in Lancang River and Guangzhou in China by Li et al. [28], Meng [37] and Xiao [59], was obviously a misidentification of *C. tilapiae* due to their high degree of similarities in both measurements and morphologies.

#### Genus *Scutogyrus* Pariselle & Euzet, 1995

##### *Scutogyrus longicornis* Paperna & Thurston, 1969

Type host: *Sarotherodon galilaeus*.

Hosts: *Sarotherodon galilaeus*, *Oreochromis mossambicus*, *Oreochromis niloticus* and *Coptodon zillii*.

Site of infection: Gills.

Type locality: Lakes Georges and Albert, Uganda.

Localities: A pond in Sun Yat-sen University and a fish farm in Guangzhou, Guangdong province; Gaozhou reservoir, Maoming, Guangdong province; Han River, Chaozhou, Guangdong Province; Xinfengjiang reservoir, Heyuan, Guangdong Province; Bachi River, Nanning, Guangxi Province; Boai River, Baise, Guangxi Province; Liu River, Liuzhou, Guangxi Province; Songtao reservoir, Danzhou, Hainan province; Shilu reservoir, Changjiang, Hainan Province; Jiatan reservoir, Chengmai, Hainan Province; Xixi River, Xiamen, Fujian Province; Jin River, Quanzhou, Fujian Province; Lancang River, Xishuangbanna, Yunnan Province; Nongba reservoir, Lincang, Yunnan Province; Lancang River, Lincang, Yunnan Province; Hualien, Taiwan.

Voucher specimens observed and deposited: 31 (SYSUSLO1–30; MNHN HEL908).

**Figure 9:**
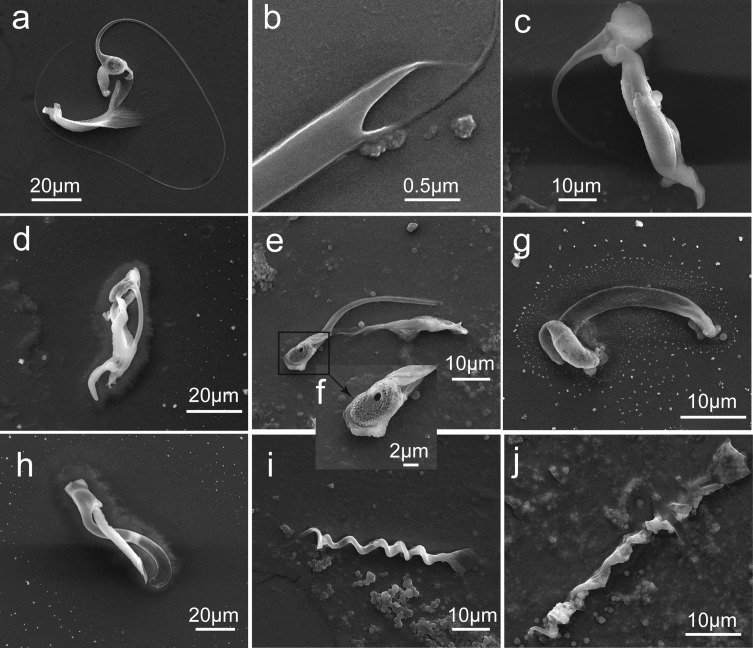
Scanning electron micrographs of genital sclerotized parts of species of *Cichlidogyrus*, *Scutogyrus* and *Enterogyrus*. (a) Male copulatory complex of *C. cirratus*; (b) penis end of male copulatory complex of *C. cirratus*; (c) male copulatory complex of *C. sclerosus*; (d) male copulatory complex of *C. thurstonae*; (e) male copulatory complex of *S. longicornis*; (f) penis basement of *S. longicornis*; (g) vagina of *S. longicornis*; (h) male copulatory complex of *C. halli*; (i) penis of *E. malmbergi*; (j) penis of *E. coronatus*.

Remarks: The description of specimens in the present study generally agrees with the previous ones of *S. longicornis* in morphologies and measurements [12, 45], except that a single large and numerous very small holes were seen on the basal portion of the male copulatory organ (see Fig. 9f) which were not previously described. This species had been reported from non-native tilapias in the Philippines [2], Mexico [23], Thailand [27], Malaysia [29], South Africa [32], Brazil [22] and China [28, 37, 58, 59].

### Family Gyrodactylidae Cobbold, 1864

#### Genus *Gyrodactylus* von Nordmann, 1832

##### *Gyrodactylus cichlidarum* Paperna, 1968

Type host: *Sarotherodon galilaeus.*

Hosts: *Oreochromis niloticus*.

Site of infection: Skin, fins and rarely gills.

Type locality: Accra plain, Ghana.

Localities: A pond in Sun Yat-sen University and a fish farm in Guangzhou, Guangdong province; Gaozhou reservoir, Maoming, Guangdong province; Songtao reservoir, Danzhou, Hainan province.

Voucher specimens observed and deposited: 13 (SYSUGCH1–13).

Remarks: The morphologies and measurements of voucher specimens in the present study agree with the previous descriptions of *G. cichlidarum* which was firstly described by Paperna [44] in Ghana and redescribed by García-Vásquez et al. [20]. This species had also been reported from non-native tilapias in the Philippines [2] (*G. niloticus* was synonymized with *G. cichlidarum* [20]) and Mexico [46].

##### *Gyrodactylus* sp1.

Hosts: *Oreochromis niloticus*.

Site of infection: Gills.

Localities: Songtao reservoir, Danzhou, Hainan province.

Voucher specimens observed and deposited: 1 (SYSUSP1-1).

**Figure 10:**
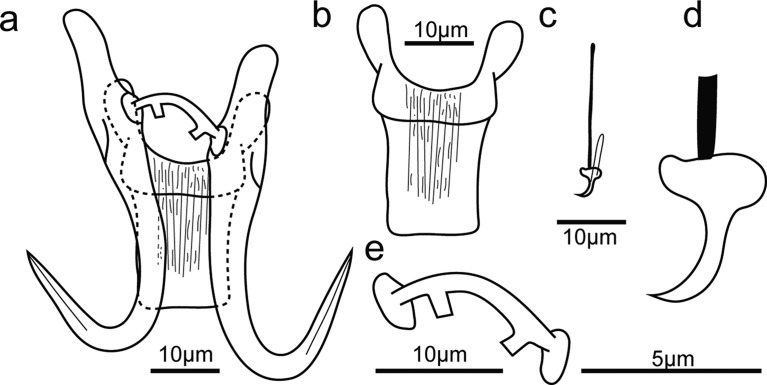
Opisthaptoral sclerotized parts of *Gyrodactylus* sp1. (a) Central hook complex; (b) ventral bar; (c) hook; (d) hook sickle; (e) dorsal bar.

Description (Fig. 10): Only one GAP mounted specimen was measured under coverslip pressure. Body 373 long, 77 wide at level of uterus. Haptor, pharynx bulb and penis not measurable. Total length of anchor (hamulus) 50, shaft 32 long, point 22 long, root 22 long, aperture distance 16, proximal shaft width 7, distal shaft width 3, inner curve length 3. Anchor aperture angle 38°, anchor point curve angle 10° and inner anchor aperture angle 43°. Dorsal bar with two protuberances, 1.4 wide, 14 long. Ventral bar with two big rounded auricular processes, 19 wide, 35 long, ventral bar processes 9 long, mid-length of ventral bar processes 12 long, median portion 5 wide, ventral bar membrane 18 long. Hooks total length 23, shaft length 18, filament loop length 11, hook aperture length 3, sickle length 4, sickle proximal width 3, sickle distal width 3, instep height 0.4 and toe length 1.2.

Remarks: Although only one specimen has been collected, its characteristics of dorsal bar (with two protuberances) and ventral bar (with two large rounded auricular processes) made it resemble *Gyrodactylus yacatli* García-Vásquez et al., 2011 [19], which was first described from the gills and fins of *O. niloticus* cultured in Mexico and also from the fins of *O. niloticus* and *Pseudocrenilabrus philander* in Zimbabwe [61]. The marginal hook sickles of the present specimen are different from former descriptions (i.e., with a larger angle) [19, 61]. However, the drawings of dorsal bar in these descriptions were not consistent; the former had a straight dorsal bar but the latter possessed a dorsal bar with two protuberances. Our specimen was more like the description of Zahradníčková et al. [61], but could not be definitively identified.

## Discussion

### Morphological characteristics of relevant monogenean species

To date, there are eight valid African species of *Enterogyrus*, namely *E. cichlidarum* Paperna, 1963; *E. malmbergi*; *E. melenensis* Bilong Bilong, Birgi & Lambert, 1989; *E. barombiensis* Bilong Bilong, Birgi & Euzet, 1991; *E. foratus* Pariselle, Lambert & Euzet, 1991; *E. coronatus*; *E. amieti* Bilong Bilong, Euzet & Birgi, 1996, and *E. crassus* Bilong Bilong, Birgi & Euzet, 1996 [3, 5–7, 49]. These species were initially described with two different opisthaptor features (cup- or tongue-shaped), which were used for the division of *Enterogyrus* into two groups [6, 30, 50]. However, the results in the present study, based on the observation of live worms of *E. coronatus* and *E. malmbergi in situ*, did not support this hypothesis. *Enterogyrus coronatus* presented a variable opisthaptoral shape during anchoring on the stomach wall (opisthaptor tongue-shaped) and shifting from one location to another (opisthaptor cup-shaped). The opisthaptor of *E. coronatus* was more variable than that of *E. malmbergi* which could only present a cup-shaped opisthaptor. In addition, the opisthaptoral sclerotized parts (posterior hooks (pairs I and II), ventral anchors and ventral bar) of *E. coronatus* were discernibly slenderer than those of *E. malmbergi*. This might facilitate the extension of the opisthaptor and its penetration into the stomach wall. Pathologically, *E. malmbergi* caused larger wounds than *E. coronatus* [31] in the stomach of the host, which might be ascribed to the larger body size and less extensible opisthaptor. This inference needs further confirmation by comparing the pathologies of other *Enterogyrus* species with slenderer opisthaptor sclerotized parts (*E. cichlidarum*, *E. melenensis*, *E. barombiensis*, *E. foratus* and *E. amieti*), and that of *E. crassus* which possesses larger opisthaptoral sclerotized parts.

The identification of *Cichlidogyrus* species was primarily based on the two-dimensional morphologies of the sclerotized parts in the whole-mount specimens, e.g., GAP preserved specimens. Based on the three-dimensional morphologies of the accessory piece terminal of the male copulatory complex of *C. cirratus*, considerable change was detected as a result of different view angle in the present study. This hints that three-dimensional morphologies of isolated sclerotized parts by modern technical methods such as laser scanning confocal fluorescence microscopy can provide more comprehensive information for taxonomic studies [18, 53].

### Monogenean fauna of exotic tilapias

Gill monogeneans from introduced tilapias have been studied widely around the world [1, 2, 22, 23, 25–29, 32, 46, 52, 56, 58], whereas few reports are available about stomach [2, 23, 30, 40], skin or fins parasites [20, 46]. However, there are no reports about urinary bladder monogeneans in invasive tilapias, though *Tilapia* sp. can be infected by *Urogyrus cichlidarum* Bilong Bilong, Birgi & Euzet, 1994 in Cameroon [4].

The introduction and spread of non-indigenous tilapias could be associated with the introduction and spread of their parasites, but the parasite species richness often decreased in comparison with that in their native range [55]. For example, it was reported that the monogenean species of *O. niloticus* numbered 18 in its native range [26, 48], while in the introduced areas they numbered between 1 and 7 although the species presented were similar [1, 2, 21, 22, 26–28, 32, 44, 50, 54, 56]. The similar monogenean species composition might be related to the similarity of tilapia strains cultured in different areas, e.g., genetically improved farmed tilapia (GIFT), which was widely introduced and cultured around the world. It was ever reported once that the monogenean species were completely lost as a result of tilapia introduction [17]. In the present study, the monogenean fauna of tilapias also shows different species loss in the different locations (see Table 1), e.g., only *C. tilapiae* was found in two sites (Haikou and Gengma) and even no parasites in Macau.

This study reported ten new host records of several monogeneans on tilapias (*E. coronatus* from *Coptodon zillii* and *O. niloticus*; *E. malmbergi* from *Coptodon zillii*, *S. galilaeus* and *O. mossambicus*; *C. halli* from *Coptodon zillii* and *O. mossambicus*; *C. thurstonae* from *Coptodon zillii*; *C. cirratus* from *O. mossambicus*; *C. sclerosus* from *S. galilaeus*), which demonstrated their lower host specificity. However, the host specificities of these species were basically consistent with previous reports [35, 51]. In addition, the distinct host preference of *Enterogyrus* species (*E. malmbergi* prefers to infect *O. niloticus*; *E. coronatus* prefers to infect *Coptodon zillii*), together with the preferences of *Cichlidogyrus* and *Scutogyrus* species to *O. niloticus* in the present study, could be ascribed to the considerable intergeneric and parental care behavior differences of hosts [35].

In China, *Cichlidogyrus levequei* Pariselle & Euzet, 1996 was previously reported from *O. niloticus* [28], but it was not collected in the present study. However, the existence of this species in China is really doubtful because authors could not provide specimens and the description was too simple to judge the species [28]. In addition, *C. levequei* was recorded to be specific to the host *Coptodon coffea* which is endemic in West Africa [48].
